# Adjustment of control in the numerical Stroop task

**DOI:** 10.3758/s13421-017-0703-6

**Published:** 2017-03-23

**Authors:** Gal Dadon, Avishai Henik

**Affiliations:** Department of Psychology and the Zlotowski Center for Neuroscience, Ben-Gurion University of the Negev, Beer-Sheva, 84105 Israel

**Keywords:** Numerical Stroop task, Proportion congruency, Adjustment of control, Task conflict, Informational conflict

## Abstract

In the numerical Stroop task, participants are asked to compare the physical sizes (physical task) or numerical values (numerical task) of two digits and ignore the irrelevant dimension. Participants are unable to ignore the irrelevant dimension as indicated by facilitation and interference effects. The literature suggests that there is asymmetry in the ability to adjust control in the physical and numerical tasks. The present study examined this suggestion in two experiments in which we manipulated the proportion of neutral/congruent trials in an experimental block. In addition, we examined the effects of control adjustment on the resolution of the task and informational conflicts. Our results suggest that adjustment of control can be bidirectional and is dependent on task requirements. Moreover, it might be easier to inhibit irrelevant information than to inhibit irrelevant task activation.

The human brain is capable of automatic processing when the information is important or when we are highly proficient in a task (for different views on automaticity see Tzelgov, Henik, Sneg, & Baruch, 1996). For example, when reaching a traffic light, stopping when the light is red and driving when the light is green does not require a substantial amount of attention. In fact, we can listen to music or just think about our day while we are choosing whether to drive or to stop. In general, automatic processing saves us time and it is cost effective. Sometimes we need to control or inhibit automatic processing, for example, when the light turns green but a child tries to cross the street and we should not drive. Moreover, there are situations in which one can implicitly adjust the inhibitory control that is implemented. The current work studies adjustment of control in a numerical Stroop task.

The Stroop task is one of the most researched tasks that have been used to examine our ability to inhibit irrelevant responses and adjust control. In the color-word version of the Stroop task, participants are presented with a colored word and are asked to name the color the word is presented in and ignore the word meaning (Stroop, 1935). Reading is a relatively automatic process and thus participants cannot ignore the act of reading the word even though it is not relevant to the task-at-hand. The automaticity of reading manifests itself in slower reaction times (RTs) to incongruent trials (i.e., trials in which the word and color do not match) compared to congruent trials (i.e., trials in which the word and color match), and the difference in RT between these conditions is referred to as the congruency effect (for a review see MacLeod, 1991). Neutral trials (i.e., a string of colored letters, e.g., XXXX) serve as a baseline, with RTs for neutral trials usually falling in the middle (i.e., slower than congruent trials and faster than incongruent trials).

One way to study adjustment in control is by manipulating the proportions of the congruency conditions in the experimental block. When the proportion of non-conflicting trials (i.e., congruent and neutral trials) increases, the congruency effect is magnified (for changes in neutral trials see Tzelgov, Henik & Berger, 1992; for changes in congruent and incongruent trials see: Bugg, McDaniel, Scullin, & Braver, 2011; Cohen, Dunbar, & McClelland, 1990; Hutchison, 2011; Lindsay & Jacoby, 1994; Logan & Zbrodoff, 1979). Changes in the proportions of congruency conditions may affect expectations and in turn, affect how alert or selective a participant is in respect to various types of trials (Botvinick, Braver, Barch, Carter, & Cohen, 2001).

Control adjustment has been studied mostly by employing a color-word Stroop task (e.g., Bugg et al., 2011; Entel, Tzelgov, Bereby-Meyer, & Shahar, 2015; Hutchison, 2011; Kalanthroff, Avnit, Henik, Davelaar, & Usher, 2015). This version of the Stroop task is limited due to its simplicity and the asymmetry between the different dimensions of the stimulus. First, in the color-word Stroop task, there is only one stimulus presented in each trial. In our everyday life, we encounter complex environments that consist of several stimuli at a time. Secondly, in the color-word Stroop task, reading is extensively more automatic than naming the color and as such, usually there is no congruency effect when participants are asked to read the word and ignore the color (Stroop, 1935). Therefore, there are two questions that remain regarding the flexibility of cognitive control adjustment mechanisms. Can we generalize previous findings to a situation in which there is more than one stimulus presented at a time and to processes other than reading? In addition, is attenuation of control limited to the most automatic process (e.g., word meaning) or can it be flexible and be dependent on task requirements? The purpose of the current study is to examine these questions. In order to do so, we used a numerical version of the Stroop task.

## Bidirectional adjustment of control

Henik and Tzelgov (1982) created the numerical Stroop task (on the basis of the original task by Besner & Coltheart, 1979) in which participants were asked to compare the physical sizes or numerical values of two digits and ignore the irrelevant dimension values. Similar to the color-word version of the Stroop task, there are three different congruency conditions: congruent – the relationship between the numbers and the physical sizes match (e.g., 3 8; the numerically smaller number appears physically smaller); incongruent – the relationship between the numbers and the physical sizes do not match (e.g., 3 8; the numerically smaller number appears physically larger); and neutral – one element is constant and one changes – for the physical task, the numerical value of the two numbers is constant (e.g., 8 8). For the numerical task, the physical size of the two numbers is constant (e.g., 4 6). The numerical versions of the Stroop task are more complex than the color-word version in the sense that in each trial there are two stimuli that are presented and have to be compared.

It is important to note that there are studies in the literature that examined control adjustment when more than one stimulus was presented. For example, some researchers used the flanker task in which there were five stimuli in a given trial (Gratton, Coles, & Donchin, 1992), some presented the two dimensions of the Stroop stimulus (i.e., color and word) separately (Appelbaum, Boehler, Davis, Won, & Woldorff, 2014), and some even used a modified Stroop task in which the conflict was between a word and a picture that were presented simultaneously (Bugg & Chanani, 2011). However, even though in these experimental designs there were multiple stimuli presented in each trial, there was only one given conflict that stemmed from a combination of the stimuli. For example, dividing the elements of the stimulus in the Stroop task does not create an additional conflict – the conflict is between the color and the word, even if they are presented separately. The numerical versions of the Stroop task are different because in addition to the conflict created by comparing the two stimuli, each stimulus presented entails a possible conflict. Namely, previous findings in the literature show that RTs for one-digit large numbers (e.g., 8) written in a big font (e.g., font size 76) are faster than RTs for one-digit large numbers written in a small font (e.g., font size 40) (Tzelgov, Meyer, & Henik, 1992). Hence, an incongruent trial in these tasks (e.g., 3 8) is incongruent for each stimulus and for the relation between them. In addition, in the numerical Stroop task, both physical and numerical dimensions are processed relatively automatically. That is, there is a congruency effect in both the physical task (i.e., when participants are asked to respond to the physical sizes of the stimuli) and numerical task (i.e., when participants are asked to respond to the numerical values of the stimuli). The two congruency effects are affected by similar factors (e.g., Leibovich, Diesendruck, Rubinsten, & Henik, 2013).

There is not a lot of research that has been carried out on adjustment of control in numbers. Borgmann, Fugelsang, Ansari, and Besner (2011) used only congruent and incongruent trials and manipulated the proportion of congruent trials in the numerical Stroop task (i.e., 25% vs. 75% proportion of congruent trials in an experimental block). They reported an attenuation of control for the numerical task. Their findings indicated a bigger congruency effect in the 75% congruent condition compared to the congruency effect in the 25% congruent condition. However, the researchers did not find indication of control adjustment in the physical task (the congruency effect in the 75% congruent condition was similar to the congruency effect in the 25% congruent condition). Borgmann and colleagues suggested that information regarding physical sizes is processed earlier and more fluently than numerical value information. Because the numerical values are processed later, the interference of the numerical value is limited and adjustment of control is not evident in the physical task (this assumption was based on previous work by Schwarz & Ischebeck, 2003). It is important to note that this interpretation implies that the most automatic process (i.e., physical size) can be controlled by top-down mechanisms whereas the less automatic process (i.e., numerical values) cannot be controlled. We would like to suggest that these conclusions might be premature and stem from differences in task difficulty.

The difficulty of a task is determined by various elements; among them are the number of possibilities for each condition and the similarity between competing elements in the stimulus. We suggest that Borgmann et al. (2011) did not observe adjustment of control in the physical task due to the fact that participants could use simple association learning mechanisms (and thus their results were not due to limitation of control mechanisms or a difference in the speed of processing numerical values). Let us elaborate. A substantial amount of studies that employ the physical and numerical tasks are asymmetric in the sense that there are more possibilities for different numerical values than physical sizes (e.g., Henik & Tzelgov, 1982; Kaufmann et al., 2008, 2005; Szűcs, & Soltész, 2007). For example, Borgmann et al. used seven different digits (2–8) to create three numerical distances (1, 3, and 5). In contrast, they used only three physical sizes, meaning that in consecutive trials, one of the physical sizes of the stimulus pair was always identical to one of the sizes in the previous stimulus pair. Under these circumstances, it can be argued that the participants could respond correctly by using simple associations learning mechanisms, thereby making the use of a top-down control mechanism redundant^1^. Therefore, it is possible that the physical task in most studies (including Besner & Coltheart’s (1979) and Henik & Tzelgov’s (1982) original studies) is easier than the numerical task. This line of thinking might explain why Borgmann et al. did not find adjustment of control in the physical task.^2^ In the current experiment, we aimed to examine whether adjustment of control can act on two automatic dimensions and be dependent on context. We ran a similar task to that of Borgmann et al. but we equalized the numerical and physical judgment tasks in respect to task difficulty by balancing the number of stimulus possibilities.

## Dissociation of task and informational conflicts

Another important question is to what extent adjustment of control affects the different components of the Stroop task. The components of the congruency effect can be used to examine resolution of two conflicts – the informational conflict and the task conflict.

The informational conflict occurs when there is a conflict between the two aspects of the stimulus (between the word and ink color). This conflict can be observed in the incongruent condition (when the word and color mismatch and lead to two competing responses). By comparing RTs of incongruent trials (which entail conflict) to RTs of neutral trials (which do not entail conflict because they lead to one relevant response), we can examine the size of the informational conflict (i.e., the interference effect). The task conflict occurs when there is a conflict between tasks. This is a conflict between the task of reading (which is irrelevant but automatic) and the task of naming the font color (which is the relevant task) (MacLeod & MacDonald, 2000). Task conflict can be observed in conditions in which the stimulus consists of a word (i.e., in both congruent and incongruent conditions). By comparing RTs of congruent trials (which entail task conflict) to RTs of neutral trials (which do not entail conflict because neutral stimuli do not consist of a word), we can examine the size of the task conflict (i.e., a smaller or no facilitation effect).

Previous studies on the color-word version of the Stroop task analyzed differences in facilitation and interference and found that when conflict is not expected, it is harder to resolve both task and informational conflicts. The basic finding is that when conflict is not expected, the interference effect is magnified and the facilitation effect decreased or even reversed in some cases (congruent trials become slower than neutral trials - the reverse facilitation effect) (e.g., Entel et al., 2015; Goldfarb & Henik, 2007; Kalanthroff, Goldfarb, Usher, & Henik, 2013; Steinhauser & Hubner, 2009). A question remains regarding the generalization of these findings to tasks in which both aspects of the stimulus are processed automatically. In the current study, we investigate if and how the task and informational conflicts are affected by changes in control adjustment in the numerical Stroop task. In order to achieve this goal we conducted two experiments.

## Experiment 1

In Experiment 1, we manipulated the proportions of neutral trials between two experimental blocks thereby creating a mostly neutral block (N75) and a minimally neutral block (N25). We chose to manipulate the proportion of neutral trials (and not the proportion of congruent-to-incongruent trials as in previous studies) for two main reasons: (1) Manipulating the proportion of congruent-to-incongruent trials creates an experimental design in which the number of congruent and incongruent trials in an experimental block is different. In our study, we aimed to compare between the task and informational conflicts and so it was important that different experimental parameters could not be used as a reason for the differences between the two conflicts. (2) Most studies that used the proportion manipulation of congruent-to-incongruent trials did not compare the task and informational conflict (e.g., Blais & Bunge, 2010; Blais, Robidoux, Risko, & Besner, 2007; Jacoby, Lindsay, & Hessels, 2003; Jacoby, McElree, & Trainham, 1999; Trainham, Lindsay, & Jacoby, 1997). In our study, we wanted to be sure that we used a proportion manipulation that affected both the task and the informational conflict. Previous findings suggested that manipulating the proportion of neutral trials affected expectation of conflict for both the informational and the task conflict (Goldfarb & Henik, 2007; Henik & Tzelgov, 1982).

We expected to find adjustment of control in both the physical and numerical tasks. We also expected to get the common pattern of facilitation and a small interference in the minimally neutral condition. In contrast, in the mostly neutral condition, we predicted a smaller or absent facilitation effect and a significant interference. In regard to differences between numerical and physical tasks, there were two theoretical possibilities that lead to different patterns of results. If adjustment of control operates on two automatic processes, we expected a similar pattern of results for the physical and numerical tasks. In contrast, if adjustment of control is limited to the most automatic dimension, we predicted adjustment of control patterns in the numerical task but not in the physical task (i.e., similar to Borgmann et al., 2011).

### Method

#### Participants

Sixty-four undergraduate students at Ben-Gurion University of the Negev participated in the study (mean age 29.98 years, *SD* = 1.65). All participants had normal or corrected-to-normal vision and no attention deficits. The participants were randomly assigned to one of two tasks – physical task and numerical task – and to one of two experimental conditions: N25 – minimally neutral condition (consisting of 25% neutral trials, 37.5% congruent trials, and 37.5% incongruent trials), and N75 – mostly neutral condition (consisting of 75% neutral trials, 12.5% congruent trials, and 12.5% incongruent trials).^3^

#### Stimuli and design

The stimuli were the same as those employed by Cohen Kadosh, Henik and Rubinsten (2008; see also Leibovich et al., 2013). We had three numerical distances (1, 2, and 5), created using the numbers 1–9 with the exclusion of the number 5. Each distance had four possible number pairs as a result of each number appearing only once for each distance (see Table 1). Similar to the numerical dimension, we had three physical distances (1, 2, and 5), created using eight physical sizes. The physical sizes that we used followed Cohen-Kadosh et al.’s (2008) work and were chosen so that RTs to all the pairs of physical sizes for a given distance were equal. Each distance had four possibilities and each physical size appeared once for each distance (see Table 1). Each digit and physical size appeared an equal number of times on the left and on the right. The stimuli in the physical and numerical tasks were the same in the congruent and incongruent condition. The neutral stimuli for the physical task consisted of the same digit in two different physical sizes (e.g., in neutral trials for the pair 3–4, the stimuli were 3 3 and 4 4). The neutral stimuli for the numerical task consisted of two different digits in the same physical size (e.g., in neutral trials for the pair 3–4, the stimuli were 4 3 and 4 3). This was important in order to make sure that the statistical analysis for congruent, incongruent and neutral trials was based on the same physical sizes and it allowed having a factorial design.

**Table 1:** The different combinations of numbers and physical sizes according to distance

Distance	Number stimuli	Physical stimuli*
1	1-2, 3-4, 6-7, 8-9	40–44, 48–52, 56–60, 67–76
2	1-3, 2-4, 6-8, 7-9	40–48, 44–52, 56–67, 60–76
5	1-6, 2-7, 3-8, 4-9	40–56, 44–60, 48–67, 52–76

* Font sizes from a viewing distance of approximately 50 cm

#### Procedure

At the beginning of the experiment, participants carried out a practice block of 16 trials, after which they completed 1,600 test trials that were divided into four blocks of 400 trials each. Between the blocks, there was a short break. The proportions of neutral trials in the physical and numerical tasks were identical in the experimental blocks. In the physical task, participants were asked to compare the physical sizes of two numbers and decide which one was larger. In the numerical task, participants were asked to compare the numerical values of two numbers and decide which one was larger. The stimuli were presented at the center of the screen and the participants were instructed to respond as quickly and accurately as possible.

**Fig. 1:**
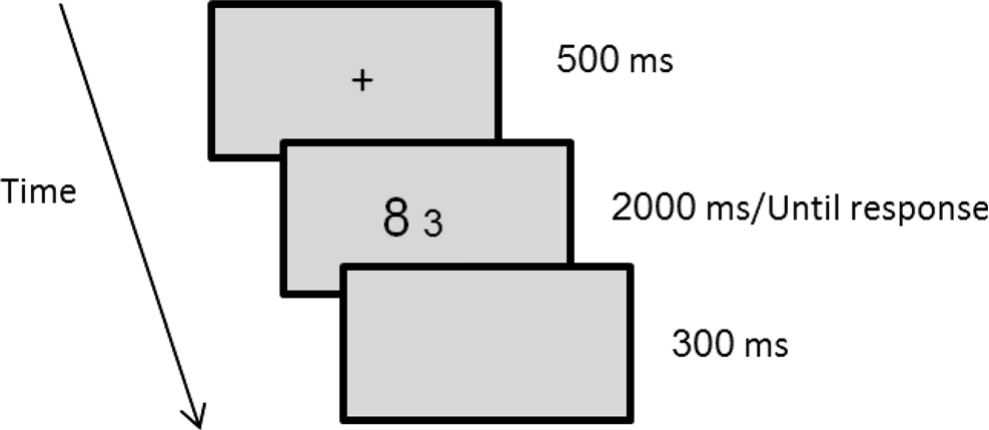
Timeline of a trial in the experiment

Each trial began with a fixation point (a plus sign) presented at the center of the screen for 500 ms, after which a pair of digits appeared and remained in view until the participant responded or 2,000 ms passed. Participants made their responses with both hands on a keyboard, by pressing one of two keys (“P” – when the right stimulus was bigger than the left stimulus, “Q” – when the left stimulus was bigger than the right stimulus). After responding. a blank screen for 300 ms and then the next trial started. The computer measured RT in milliseconds from the onset of the stimulus (see Fig. 1).

### Results

#### Error rates analysis

The data of one participant were removed from the analysis due to low accuracy rates (the participant performed 3.15 standard deviations (SDs) below the group mean accuracy, which was 93%). Calculation of the facilitation and interference effects involves comparing congruent and incongruent trials to the neutral trials. In this situation, the facilitation and interference effects are both affected by the same neutral trials and so a change in the neutral trials will inevitably cause a change in facilitation and interference. In order to avoid analysis of such dependent measures, we randomly divided the neutral trials into two groups. We used one group of neutral trials to examine the facilitation effect and the other group to examine the interference effect. Dividing the neutral trials into two groups enabled us to measure, the facilitation and interference effects independently from one another and as such, a change in the facilitation component will not immediately mean a change in the interference component. We followed this logic for all our analyses.

We conducted a 4 × 2 × 2 mixed analysis of variance (ANOVA) with congruency (congruent, neutral-congruent, neutral-incongruent and incongruent) as a within-participants factor, and proportion condition (N25 vs. N75) and task (physical task vs. numerical task) as between-participants factors. The main effect of congruency was significant, *F*(3, 177) = 119.2, *p* < .01, *η*_*p*_^2^ = .67, meaning there were significant facilitation and interference effects, *F*(1, 59) = 147.17, *p* < .01, *η*_*p*_^2^ = .71 and *F*(1, 59) = 94.68, *p* < .01, *η*_*p*_^2^ = .61, respectively. There was no difference between the two neutral conditions, *F*(1, 59) < 1, *ns*. The main effect of task was significant, *F*(1, 59) = 6.74, *p* < .01, *η*_*p*_^2^ = .1, meaning error rates for the physical task were higher than error rates for the numerical task. Importantly, the 3-way interaction was not significant, *F*(3, 177) < 1, *ns*, and the two 2-way interactions were significant.

**Fig. 2:**
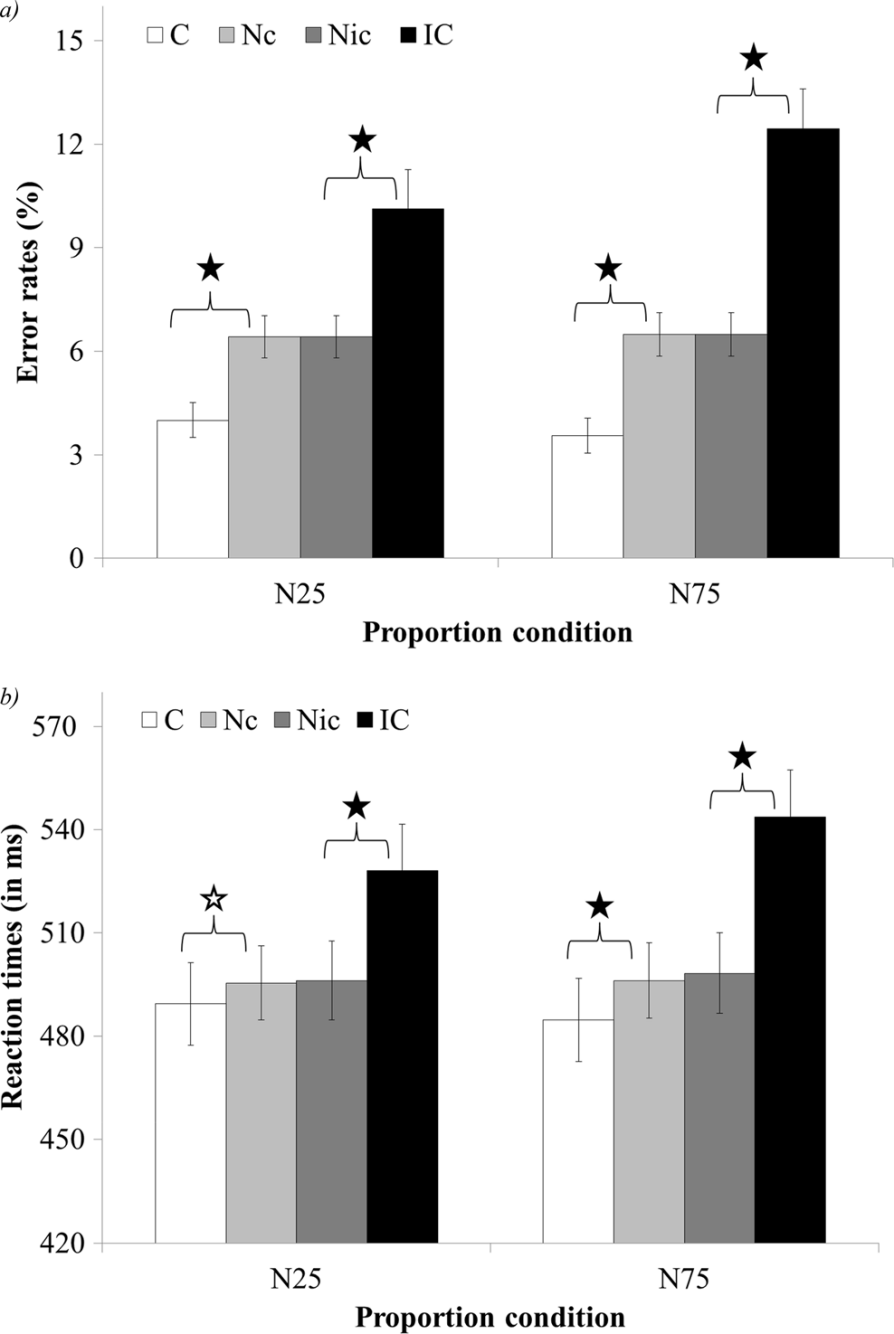
Error rates (**a**) and reaction times (**b**) of the congruency and proportion condition interaction in Experiment 1. X axis – proportion of neutral trials in a block. *N25* minimally neutral block, *N75* mostly neutral block, *C* congruent, *Nc* neutral trials that were in the comparison between neutral and congruent trials, *Nic* neutral trials that were in the comparison between neutral and incongruent trials, *IC* incongruent. *significant result, *p* < .01; ✩– marginally significant

The interaction between the congruency (congruent, neutral-congruent, neutral-incongruent, and incongruent) and proportion condition (N25 vs. N75) was significant, *F*(3, 177) = 4.61, *p* < .01, *η*_*p*_^2^ = .07 (see Fig. 2a). Planned comparison revealed a larger congruency effect in N75 compared to the N25 condition, *F*(1, 59) = 4.83, *p* < .05 *η*_*p*_^2^ = .07.^4^ Analysis of the facilitation and interference effects revealed that the facilitation effect was similar in the two proportion conditions, *F*(1, 59) = 1.14, *ns* (i.e., the facilitation was significant in both N25 and N75, *F*(1, 59) = 60.91, *p* < .01, *η*_*p*_^2^ = .51 and *F*(1, 59) = 87.23 *p* < .01, *η*_*p*_^2^ = .6, respectively). In addition, the interference effect was larger in the N75 condition compared to the N25 condition, *F*(1, 59) = 5.06, *p* < .05, *η*_*p*_^2^ = .08.

**Fig. 3:**
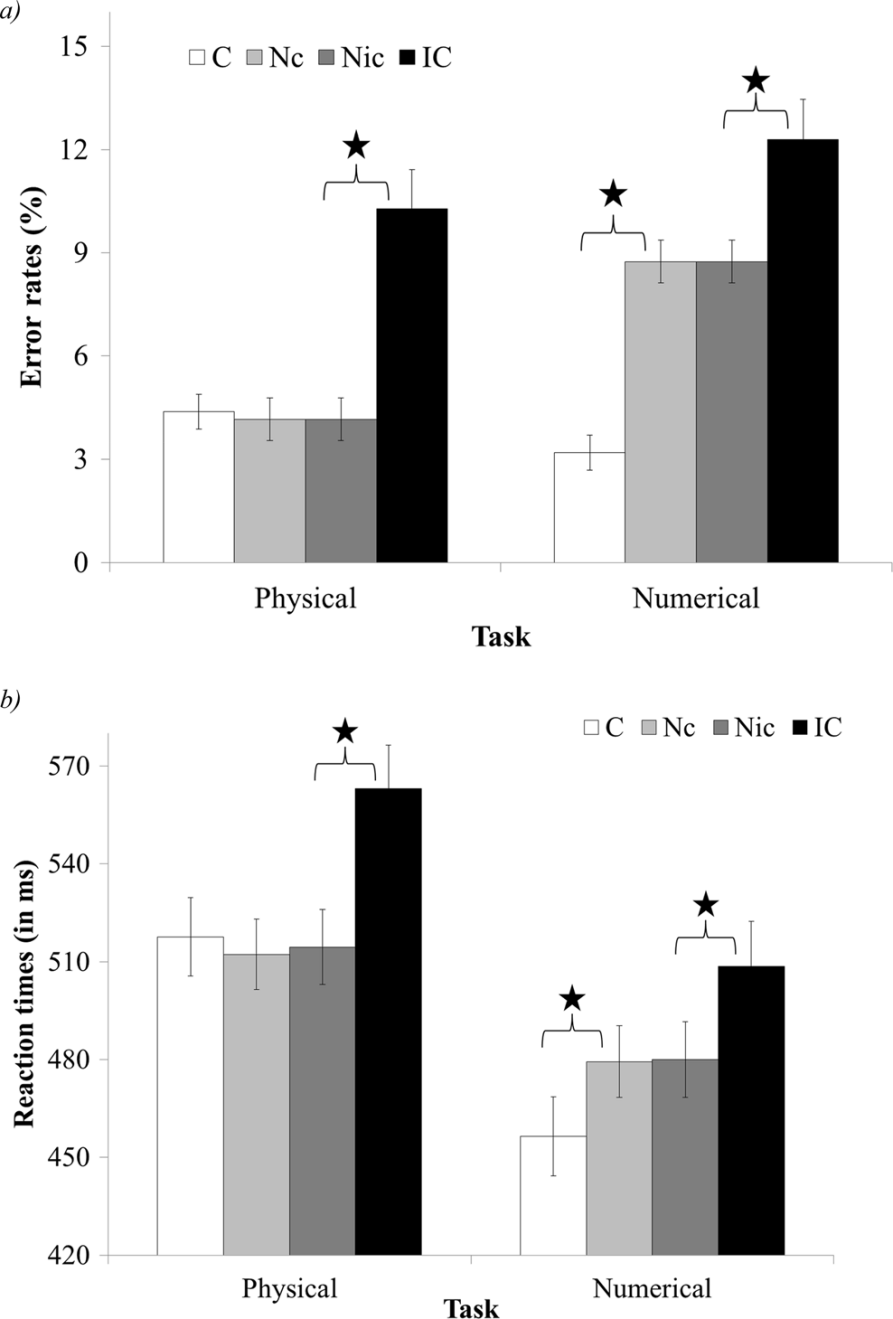
Error rates (**a**) and reaction times (**b**) of the congruency and task interaction in Experiment 1. *C* congruent, *Nc* neutral trials that were in the comparison between neutral and congruent trials, *Nic* neutral trials that were in the comparison between neutral and incongruent trials, *IC* incongruent. *significant result, *p* < .01

The 2-way interaction between congruency (congruent, neutral-congruent, neutral-incongruent, and incongruent) and task (physical task vs. numerical task) was significant, *F*(3, 177) = 22.88, *p* < .01, *η*_*p*_^2^ = .28 (see Fig. 3a). Planned comparisons revealed a larger congruency effect in the numerical task compared to the physical task, *F*(3, 177) = 22.88, *p* < .01, *η*_*p*_^2^ = .1. In addition, there was a significant difference in the facilitation and interference components between tasks, *F*(1, 59) = 172.31, *p* < .01, *η*_*p*_^2^ = .74 and *F*(1, 59) = 6.71, *p* < .05, *η*_*p*_^2^ = .1, respectively. Namely, there was a significant facilitation in the numerical task, *F*(1, 59) = 313.84, *p* < .01, *η*_*p*_^2^ = .84, but no facilitation in the physical task, *F*(1, 59) < 1, *ns*. In addition, there was a larger interference in the physical task compared to the numerical task, *F*(1, 59) = 6.71, *p* < .05, *η*_*p*_^2^ = .1.

#### Reaction time analysis

We conducted a 4 × 2 × 2 mixed ANOVA with congruency (congruent, neutral-congruent, neutral-incongruent, and incongruent) as a within-participants factor, and proportion condition (N25 vs. N75) and task (physical task vs. numerical task) as between-participants factors. The main effect of congruency was significant, *F*(3, 177) = 137.76, *p* < .01, *η*_*p*_^2^ = .7, meaning there were significant facilitation and interference effects, *F*(1, 59) = 12.34, *p* < .01, *η*_*p*_^2^ = .17 and *F*(1, 59) = 172.09, *p* < .01, *η*_*p*_^2^ = .74, respectively. There was no difference between the two neutral conditions, *F*(1, 59) < 1, *ns*. The main effect of task was significant, *F*(1, 59) = 7.48, *p* < .01, *η*_*p*_^2^ = .12, meaning RTs for the physical task were slower than RTs for the numerical task. Importantly, the 3-way interaction was not significant, *F*(3, 177) < 1, *ns*, and the two 2-way interactions were significant.

The interaction between the congruency (congruent, neutral-congruent, neutral-incongruent, and incongruent) and proportion condition (N25 vs. N75) was significant, *F*(3, 177) = 5.41 *p* < .01, *η*_*p*_^2^ = .08 (see Fig. 2b). Planned comparison revealed a larger congruency effect in N75 compared to the N25 condition, *F*(1, 59) = 12.46, *p* < .01 *η*_*p*_^2^ = .17. Analysis of the facilitation and interference effects revealed that the facilitation effect was similar in the two proportion conditions, *F*(1, 59) = 1.13, *ns*. It is important to note that the facilitation effect in the N25 condition was only marginally significant, *F*(1, 59) = 3.04, *p* = .09, *η*_*p*_^2^ = .05, and as such, the facilitation effect mostly stemmed from the N75 condition in which the facilitation was significant, *F*(1, 59) = 10.32, *p* < .01, *η*_*p*_^2^ = .15. In addition, the interference effect was larger in the N75 condition compared to the N25 condition, *F*(1, 59) = 5.21, *p* < .05, *η*_*p*_^2^ = .08.

The 2-way interaction between congruency (congruent, neutral-congruent, neutral-incongruent, and incongruent) and task (physical task vs. numerical task) was significant, *F*(3, 177) = 14.74, *p* < .01, *η*_*p*_^2^ = .2 (see Fig. 3b). Planned comparisons revealed that there was no difference in the general size of the congruency effect between tasks, *F*(1, 59) = 1.44, *ns*, but there was a significant difference in the facilitation and interference components between tasks. Namely, there was a significant facilitation in the numerical task, *F*(1, 59) = 41.48, *p* < .01, *η*_*p*_^2^ = .41, but no facilitation in the physical task, *F*(1, 59) = 2.36, *ns*. Given the large *F* value, it should be noted that the trend of facilitation in the physical task (although not significant) was that of a reverse facilitation (the congruent trials were slower than the neutral trials). In addition, there was larger interference in the physical task compared to the numerical task, *F*(1, 59) = 11.39, *p* < .01, *η*_*p*_^2^ = .16.

### Discussion of experiment 1

The results of Experiment 1 revealed similar patterns of control adjustment for the physical and numerical tasks. In both the error rates and the RTs analyses, there was a larger interference effect in the mostly neutral block compared to the minimally neutral block. The latter indicates a decline in the ability to resolve the informational conflict when there is a large proportion of neutral trials. We did not find control adjustment for the task conflict; namely, there was a similar facilitation effect in the mostly neutral block and the minimally neutral block.

As was discussed in the introduction, the rationale behind our design was that manipulating neutral trials is a cleaner manipulation than manipulating the proportion of congruent-to-incongruent trials. However, it should be noted that our design was different from Borgmann et al.’s (2011) design in two main aspects (i.e., the stimuli and the proportion manipulation). These differences can lead to two alternative explanations for our results. The first explanation is that the triple interaction found in Borgmann et al.’s study was due to changes in task difficulty. In our task, the task difficulty was equalized and as such, the triple interaction was not significant. In contrast, it could be claimed that manipulating the proportion of neutral trials is inherently different from manipulating the proportion of congruent-to-incongruent trials and as a result, the lack of the triple interaction was due to this change in our design.

In order to rule out the alternative explanation regarding the difference between manipulating the proportion of neutral trials compared to manipulating the proportion of congruent-to-incongruent trials, we designed Experiment 2. In Experiment 2, we manipulated the proportions of congruent-to-incongruent trials while using the same stimuli as in Experiment 1.

## Experiment 2

### Method

The method of Experiment 2 was identical to that of Experiment 1 except that in Experiment 1 we manipulated the proportion of neutral trials while in Experiment 2 we manipulated the proportion of congruent-to-incongruent trials (in Experiment 2 the number of neutral trials remained constant between conditions). Sixty-four undergraduate students (mean age 23.08 years, *SD* = 1.32), who fit the same restrictions that were described in Experiment 1, participated in the study. The participants were randomly assigned to one of two tasks – the physical task or the numerical task – and to one of two experimental conditions: C25 – minimally congruent condition and C75 – mostly congruent condition.

### Results

#### Error rates analysis

Three participants (from three different experimental groups) were removed from the analysis due to low accuracy rates (the participants performed 3.1, 3.4, and 4.8 SDs below the group mean accuracy, which was 93%).

Similar to Experiment 1, we randomly divided the neutral trials into two groups. We used one group of neutral trials to examine the facilitation effect and the other group to examine the interference effect. We conducted a 4 × 2 × 2 mixed ANOVA with congruency (congruent, neutral-congruent, neutral-incongruent, and incongruent) as a within-participants factor, and proportion condition (C25 vs. C75) and task (physical task vs. numerical task) as between-participants factors. The main effect of congruency was significant, *F*(3, 171) = 89.81, *p* < .01, *η*_*p*_^2^ = .61, meaning there were significant facilitation and interference effects, *F*(1, 57) = 71.1, *p* < .01, *η*_*p*_^2^ = .55 and *F*(1, 57) = 71.62, *p* < .01, *η*_*p*_^2^ = .56, respectively. There was no difference between the two neutral conditions, *F*(1, 57) < 1, *ns*. The main effect of task was not significant, *F*(1, 57) < 1, *ns*, meaning that the error rates in the physical and the numerical task were similar. Importantly, the 3-way interaction was not significant, *F*(3, 171) = 1.72, *ns*, and the two 2-way interactions were significant.

**Fig. 4:**
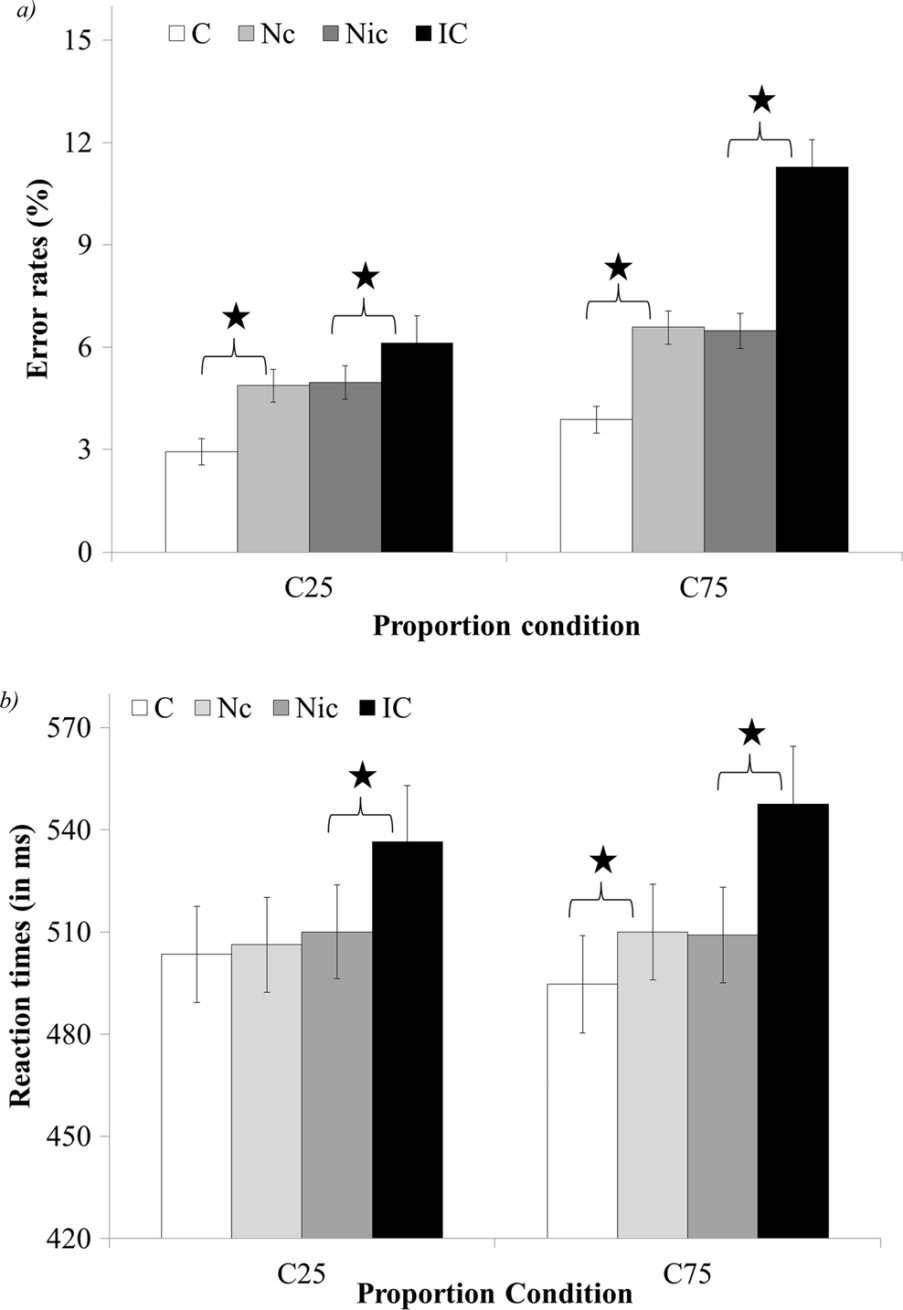
Error rates (**a**) and reaction times (**b**) of the congruency and proportion condition interaction in Experiment 2. X axis – proportion of neutral trials in a block. *C25* minimally neutral block, *C75* mostly neutral block, *C* congruent, *Nc* neutral trials that were in the comparison between neutral and congruent trials, *Nic* neutral trials that were in the comparison between neutral and incongruent trials, *IC* incongruent. *significant result, *p* < .01

The interaction between congruency (congruent, neutral-congruent, neutral-incongruent, and incongruent) and proportion condition (C25 vs. C75) was significant, *F*(3, 177) = 17.45 *p* < .01, *η*_*p*_^2^ = .23 (see Fig. 4a). Planned comparison revealed a larger congruency effect in C75 compared to the C25 condition, *F*(1, 57) = 20.2, *p* < .05, *η*_*p*_^2^ = .26. Analysis of the facilitation and interference effects revealed that the facilitation effect was similar in the two proportion conditions, *F*(1, 57) = 1.91, *ns* (the facilitation was significant in both C25 and C75, *F*(1, 57) = 25.25, *p* < .01, *η*_*p*_^2^ = .31 and *F*(1, 57) = 47.4, *p* < .01, *η*_*p*_^2^ = .45, respectively). In addition, the interference effect was larger in the C75 condition compared to the C25 condition, *F*(1, 57) = 26.9, *p* < .01, *η*_*p*_^2^ = .5.

**Fig. 5:**
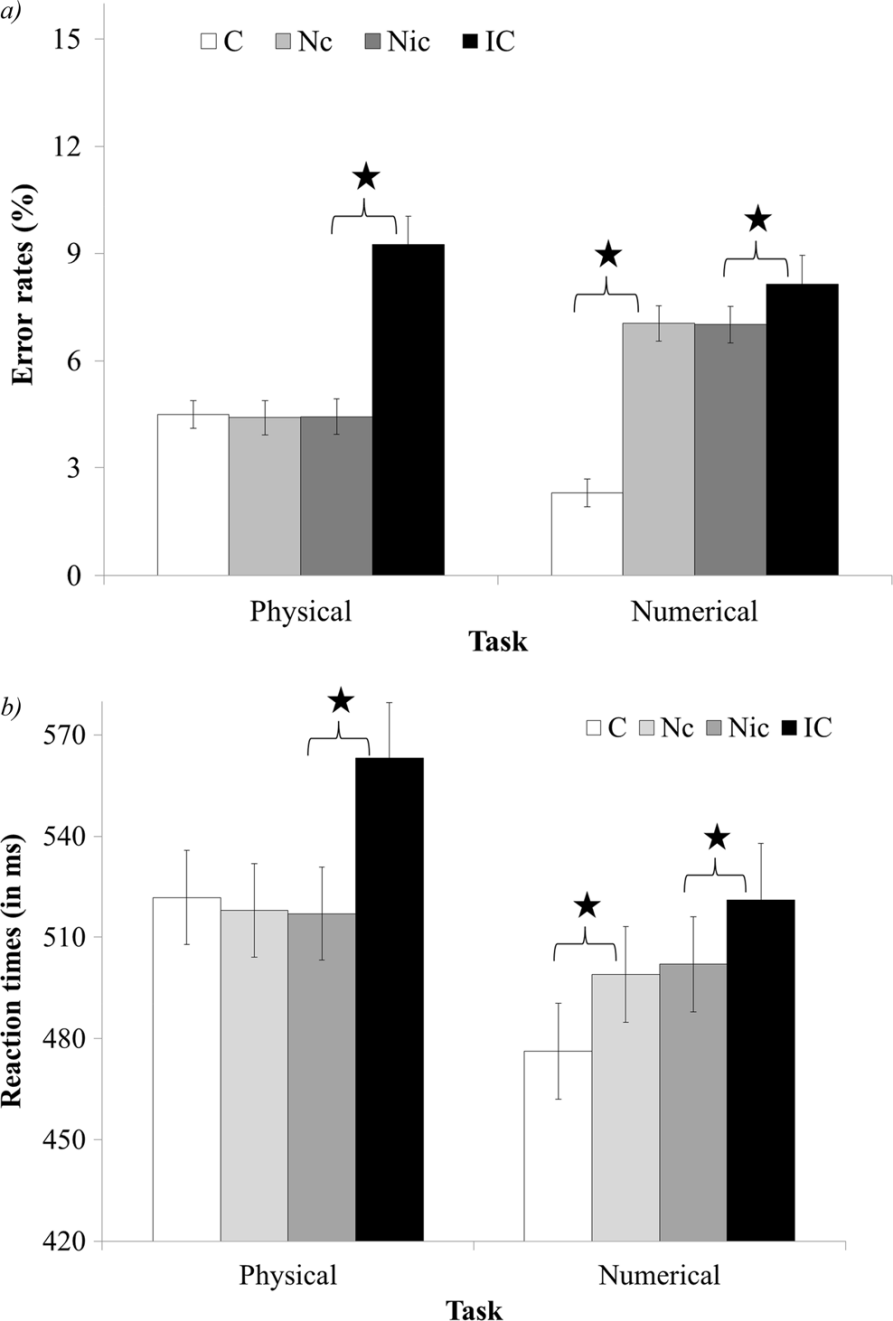
Error rates (**a**) and reaction times (**b**) of the congruency and task interaction in Experiment 2. *C* congruent, *Nc* neutral trials that were in the comparison between neutral and congruent trials, *Nic* neutral trials that were in the comparison between neutral and incongruent trials, *IC* incongruent. *significant result, *p* < .01

The 2-way interaction between congruency (congruent, neutral-congruent, neutral-incongruent, and incongruent) and task (physical task vs. numerical task) was significant, *F*(3, 171) = 29.74, *p* < .01, *η*_*p*_^2^ = .34 (see Fig. 5a). Planned comparisons indicated there was a similar congruency effect in the numerical and physical tasks, *F*(1, 57) = 1.33, *ns*. The components of the congruency effect (the facilitation and interference) changed significantly between tasks, (*F*(1, 57) = 76.96, *p* < .01, *η*_*p*_^2^ = .57 and *F*(1, 57) = 27.46, *p* < .01, *η*_*p*_^2^ = .32, respectively). Namely, there was a significant facilitation in the numerical task, *F*(1, 57) = 145.69, *p* < .01, *η*_*p*_^2^ = .72, but no facilitation in the physical task, *F*(1, 57) < 1, *ns*. In addition, there was a larger interference in the physical task compared to the numerical task.

#### Reaction times analysis

We conducted a 4 × 2 × 2 mixed ANOVA with congruency (congruent, neutral-congruent, neutral-incongruent, and incongruent) as a within-participants factor, and proportion condition (C25 vs. C75) and task (physical task vs. numerical task) as between-participants factors. The main effect of congruency was significant, *F*(3, 171) = 109.22, *p* < .01, *η*_*p*_^2^ = .66, meaning there were significant facilitation and interference effects, *F*(1, 57) = 15.08, *p* < .01, *η*_*p*_^2^ = .21 and *F*(1, 57) = 132.72, *p* < .01, *η*_*p*_^2^ = .7, respectively. There was no difference between the two neutral conditions, *F*(1, 57) < 1, *ns*. The main effect of task was not significant, *F*(1, 57) = 2.18, *ns*, meaning RTs for the physical task and RTs for the numerical task were similar. Importantly, the 3-way interaction was not significant, *F*(3, 171) < 1, *ns*, and the two 2-way interactions were significant.

The interaction between congruency (congruent, neutral-congruent, neutral-incongruent, and incongruent) and proportion condition (C25 vs. C75) was significant, *F*(3, 171) = 5.28, *p* < .01, *η*_*p*_^2^ = .08 (see Fig. 4b). Planned comparison revealed a larger congruency effect in C75 compared to the C25 condition, *F*(1, 57) = 9.92, *p* < .01 *η*_*p*_^2^ = .15. Analysis of the facilitation effect revealed a significant difference between proportion conditions, *F*(1, 57) = 7.04, *p* < .01, *η*_*p*_^2^ = .1. Namely, the facilitation effect was significant in the C75 condition, *F*(1, 57) = 21.03, *p* < .01, *η*_*p*_^2^ = .27, but not significant in the C25 condition, *F*(1, 57) < 1, *ns*. The interference effect was significantly larger in the C75 condition compared to the C25 condition, *F*(1, 57) = 4.08, *p* < .01, *η*_*p*_^2^ = .07.

The 2-way interaction between congruency (congruent, neutral-congruent, neutral-incongruent, and incongruent) and task (physical task vs. numerical task) was significant, *F*(3, 171) = 18.57, *p* < .01, *η*_*p*_^2^ = .25 (see Fig. 5b). Planned comparisons indicated there was a similar congruency effect in the numerical and physical tasks, *F*(1, 57) < 1, *ns*. Comparisons of the facilitation and interference effects revealed that there was significant facilitation in the numerical task, *F*(1, 57) = 42.14, *p* < .01, *η*_*p*_^2^ = .44, but no facilitation in the physical task, *F*(1, 57) = 1.66, *ns*. It should be noted that similar to Experiment 1, the large *F* value was due to a not significant tendency to a reverse facilitation in the physical task (the congruent trials were slower than the neutral trials). In addition, there was larger interference in the physical task compared to the numerical task, *F*(1, 57) = 22.33, *p* < .01, *η*_*p*_^2^ = .28.

### Discussion of experiment 2

In general, the results of Experiment 2 replicated the pattern of results found in Experiment 1. The similarity between experiments suggests that the task difficulty and not the difference in the proportion manipulation was the cause for the lack of the triple interaction in our study. We found similar patterns of control adjustment for the physical and numerical tasks. In both the error rates and the RTs analyses there was a larger interference effect in the mostly congruent block compared to the minimally congruent block. The latter indicates a decline in the ability to resolve the informational conflict when there was a large proportion of congruent trials. We did not find control adjustment for the task conflict in the error rates analysis (there was a similar facilitation effect in the mostly congruent block and the minimally congruent block). However, unlike in Experiment 1, we found a significant facilitation in the mostly congruent block compared to the minimally congruent block.

We suggest that the difference in the facilitation effect in RT analysis between Experiments 1 and 2 might be due to two main reasons. The first reason relates to differences in differential practice that were a possible confound in Experiment 2 but not in Experiment 1. Namely, in Experiment 2 the number of congruent and incongruent trials changed within each condition. In this design, the finding of a facilitation only in RTs and only in a block in which the congruent trials were the most practiced trials might be less convincing. This possible explanation emphasizes the importance of manipulating the proportion of neutral trials in which the amount of differential practice for congruent and incongruent trials is equal within each experimental condition.

A second explanation for the differences between the results of Experiments 1 and 2 might be related to the different nature of the proportion manipulation between the experiments. Namely, the neutral trials used in our experiment were trials in which there was no task conflict and no informational conflict. By manipulating the proportion of neutral trials, we manipulated the expectation of conflict for both the task and the informational conflict equally. In contrast, in the congruent trials, there was a task conflict (but not an informational conflict). Hence, manipulation of the proportion of congruent-to-incongruent trials created a different expectation of conflict for the task and the informational conflicts. This difference in the nature of the proportion manipulation could explain the differences in the pattern of results. Further research is needed in order to examine this hypothesis.

## General discussion

In the current study, we aimed to examine three questions regarding the flexibility of control adjustment mechanisms and the way these mechanisms modulate task and informational conflicts. Our questions were as follow: (1) Can previous findings regarding top-down control adjustment be generalized to tasks that involve processing of magnitudes and in which there is more than one stimulus (and more than one conflict) presented at a time? (2) Are control adjustment mechanisms limited to the most automatically processed dimension? (3) What is the relationship between control adjustment mechanisms and the informational and task conflicts?

In order to examine these questions, we manipulated the proportion of neutral trials (Experiment 1) and the proportion of congruent-to-incongruent trials (Experiment 2) in the numerical Stroop task. In addition, we controlled for differences in task difficulty between the physical and numerical tasks. We found similar patterns of control adjustment in the physical and numerical tasks for both experiments. There was a larger interference effect in conditions in which conflict was not expected (mostly neutral block and mostly congruent block) compared to conditions in which conflict was expected (minimally neutral block and minimally congruent block). We did not find control adjustment for the task conflict in any of the analyses except for one; namely, expectation of conflict did not change the size and direction of the facilitation effect. The exception to this rule was the finding of a larger significant facilitation effect in the RTs of the mostly congruent block compared to the minimally congruent block (Experiment 2).

Our line of results suggests that top-down control mechanisms operate in similar ways regardless of the task. Most studies examined top-down mechanisms by examining the ability to inhibit reading of a single colored word, or examining several stimuli in which there was only one conflict that stemmed from the combination of all stimuli (i.e., Appelbaum et al., 2014; Bugg et al., 2011; Cohen et al., 1990; Entel et al., 2015; Gratton et al., 1992; Hutchison, 2011; Kalanthroff et al., 2015; Lindsay & Jacoby, 1994; Logan & Zbrodoff, 1979; Steinhauser & Hubner, 2009; Tzelgov, Henik et al., 1992). In our study, we used the numerical Stroop task in which participants were asked to compare the magnitude of numbers or their physical sizes. The existence of top-down modulation we found in the numerical Stroop task demonstrates that previous findings can be extended to a somewhat more complex environment (with two stimuli and several conflicts) and to processes other than reading (processing of numbers and physical sizes).

In the common color-word Stroop task, one dimension is processed much more automatically – the word dimension (Stroop, 1935). In contrast, in the numerical versions of the Stroop task, both dimensions (numerical value and physical size) are processed relatively automatically and the relevant and irrelevant dimensions change according to task (e.g., numbers are relevant in the numerical task and irrelevant in the physical task) (Henik & Tzelgov, 1982). The fact that we found similar patterns of control adjustment in the physical and numerical tasks suggests that top-down control mechanisms can take into account task requirements and are not limited to the mostly automatic dimension.

The third goal of our study was to investigate the relationship between control adjustment mechanisms and the components of the Stroop effect. The magnitude of interference and facilitation effects is modulated by our ability to resolve the informational and task conflict (e.g., Entel et al., 2015; Goldfarb & Henik, 2007; Kalanthroff, Goldfarb, & Henik, 2013; Kalanthroff, Goldfarb, Usher et al., 2013; Kalanthroff & Henik, 2014). The informational conflict represents the ability to inhibit irrelevant information, and the task conflict represents the ability to inhibit irrelevant tasks (Goldfarb & Henik, 2007; MacLeod, & MacDonald, 2000). Understanding the extent to which top-down control mechanisms affect different conflicts contributes to our understanding of the flexibility of cognitive control mechanisms. Findings in the literature regarding the color-word Stroop task suggested similar adjustment of control modulation for informational and task conflict (e.g., Entel et al., 2015; Goldfarb & Henik, 2007; Kalanthroff & Henik, 2014). In contrast, the current study demonstrated an asymmetry between the informational and task conflicts. Specifically, we observed top-down control adjustment for the interference effect but in general, this effect did not appear for the facilitation effect. It is important to note that Kalanthroff, Goldfarb and Henik (2013) showed dissociation between the task and informational conflict but from a different perspective. By combining a color-word Stroop task with a stop-signal task, they demonstrated modulation of the task conflict but not the informational conflict. Both our study and the one of Kalanthroff, Goldfarb and Henik (2013) support the notion that task and informational conflicts involve different control mechanisms. Hence, in the numerical Stroop task it might be easier to adjust the amount of control invested in inhibiting irrelevant information (attenuation of the informational conflict) than to adjust the amount of control invested in inhibiting irrelevant tasks (attenuation of the task conflict).

The differences between the two versions of the Stroop task (the color-word version and the numerical versions) in the ability to modulate the task conflict might stem from the nature of the stimulus in each of the tasks. In the color-word Stroop task, the irrelevant task is significantly different from the relevant task (reading is different from processing and naming a color). In contrast, in the numerical Stroop task, the relevant and irrelevant tasks are similar in the sense that both tasks involve comparison of magnitudes. Moreover, it was found that both the numerical and physical tasks activate similar brain areas (for a review see Cohen Kadosh, Lammertyn, & Izard, 2008; see also Cohen Kadosh & Henik, 2006a, 2006b; Kaufmann et al., 2005; Pinel, Piazza, La Bihan, & Dehaene, 2004; Schwarz & Heinze, 1998). It is possible that in situations where there are two tasks similar in nature, there is a constant need for an active control mechanism to inhibit the irrelevant task. Therefore, it is harder to “put the guard to sleep” and modulate the task conflict.^5^ This hypothesis could explain why in the color-word Stroop task there is modulation of both task and informational conflict whereas in the numerical Stroop task no modulation of the task conflict is observed. Further research is needed in order to examine this hypothesis.

The current results can also broaden the scope of previous findings in respect to the way we examine automaticity of processing symbolic numbers and physical sizes. Most studies that employ the numerical Stroop task do so in a way that creates an asymmetry in the difficulty of the physical and numerical tasks. The physical task is easier and so the mean RT in physical comparisons is faster in comparison to the numerical task (e.g., Henik & Tzelgov, 1982; Kaufmann et al., 2008, 2005; Szűcs & Soltész, 2007). Borgmann et al. (2011) were aware of the asymmetry in task difficulty between the physical and numerical tasks and tried to overcome this potential confound. They did so by selecting more difficult physical sizes that were chosen based on matching RTs and error rates in comparisons of numerical values. These researchers suggested that by comparing adjustment of control in the physical and numerical task, one could examine which of the two processes was more basic and automatic. They manipulated the proportion of congruent trials in a common numerical Stroop task (i.e., 25% congruent trials vs. 75% congruent trials in an experimental block) and compared attenuation of control in the physical and numerical task. They reported adjustment of control for the numerical task but not for the physical task. Borgmann and colleagues interpreted this result by suggesting that information of physical sizes is processed before information of numerical values, and that is why the interference of the numerical value is limited (their assumption was based on previous work by Schwarz & Ischebeck, 2003). Our results contradict Borgmann et al.’s findings. We found attenuation of control in both the physical and numerical task, which suggests that processing of numerical values can be as automatic as processing of physical sizes. We would like to suggest that the contradiction between the two studies could be explained by a difference in task difficulty. This difference existed in the original work by Henik and Tzelgov (1982). It is built into the design of the common numerical Stroop task and it was not controlled in Borgmann et al.’s study. We discuss this issue in more details in the following paragraph.

A significant difference between our study and Borgmann et al.’s (2011) study is the way we equalized the task difficulty between the physical and numerical tasks. Borgmann et al. controlled task difficulty by equating the mean RT for the tasks. In contrast, we controlled task difficulty by balancing the number of stimulus possibilities between tasks.^6^ Recently, Bugg (2014) suggested the AATC (associations as antagonists to top-down control) model, which states that top-down control mechanisms will be activated only in tasks in which it is difficult to use simple associative learning mechanisms. One implication of the AATC model is that balancing task difficulty by equating mean RTs cannot eliminate participants’ ability to use simple association learning mechanisms. Although in Borgmann et al.’s study the RTs of the physical and numerical tasks were similar, the number of different stimuli possibilities in the physical task was significantly lower compared to in the numerical task (there were only three physical sizes). Hence, the physical task was easy in the sense that participants could use simple association learning mechanisms. Top-down adjustment mechanisms were not needed and that is why no attenuation of control was observed for the physical task. In contrast, in our study the number of possibilities for physical sizes was significantly larger and similar to the number of possibilities for numerical values. Hence, participants could not respond according to simple associations and that is why top-down control modulation was observed. The comparison between the two studies can provide further support for the AATC model and presents evidence that processing of numerical values can be automatic, similar to the processing of physical sizes.

To conclude, we suggest that adjustment of control can be bidirectional and dependent on task requirements. Moreover, it might be easier to inhibit irrelevant information than to inhibit irrelevant task activation when the relevant and irrelevant tasks are similar in nature. In addition, we suggest that cognitive control mechanisms can prioritize processing of the numerical dimension relative to the physical dimension and that different findings in the literature regarding processing speed of numbers and physical sizes can be due in part to differences in task difficulty.

## Footnotes

1 This suggestion is in line with Bugg’s (2014) AATC model. According to the model, top-down control mechanisms are activated only in tasks in which it is difficult to use simple associative-learning mechanisms.

2 It is important to note that Borgmann et al. (2011) attempted to equalize task difficulty by matching the RTs and error rates of the physical and numerical tasks. However, this kind of balancing of task difficulty cannot eliminate a participant's ability to use simple association-learning mechanisms.

3 These proportions were chosen to be equivalent to "Tzelgov et al.’s (1992)" was link to "Tzelgov, Meyer, et al., (1992)". Please check if correct.Tzelgov et al.’s (1992) study on attenuation of control in the color-word Stroop task.

4 It is important to note that a previous study by Jonides and Mack (1984) pointed out some problems that might be relevant to analyses in which neutral trials are used. Hence, we added an analysis of the congruency effect in order to make sure that the basic pattern of control adjustment exist even without using the neutral trials in the analysis.

5 The metaphor “putting the guard to sleep” was first described in Goldfarb and Henik’s (2007) study and it is used to describe behavior in situations in which conflict is not expected.

6 This issue is discussed extensively in the introduction and the method sections.
